# Supplementary figures and images for: Optical Recordings of Unitary Synaptic Connections Reveal High and Random Local Connectivity between CA3 Pyramidal Cells
Source: J Neurosci. 2025 Jul 24;45(36):e0102252025. doi: 10.1523/JNEUROSCI.0102-25.2025 (PMC12410047; doi:10.1523/JNEUROSCI.0102-25.2025)

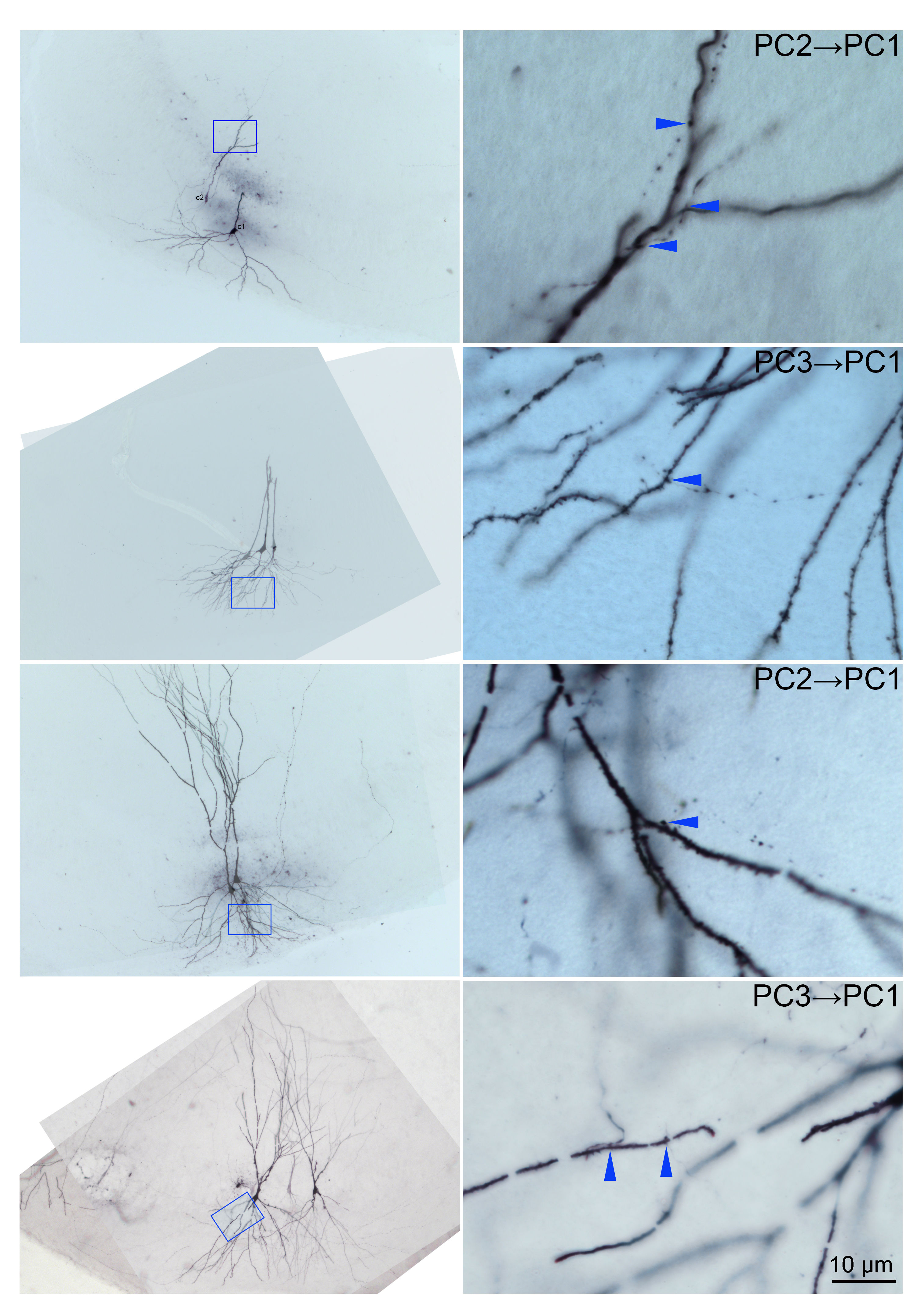

Supplement: Figure 1-1 — Identification of contact sites between axons and dendrites of biocytin-labeled CA3PCs in DAB-stained samples. Scale bar for all high magnification images: 10 µm. Download Figure 1-1, TIF file. [file jneuro-45-e0102252025-s001.tif]

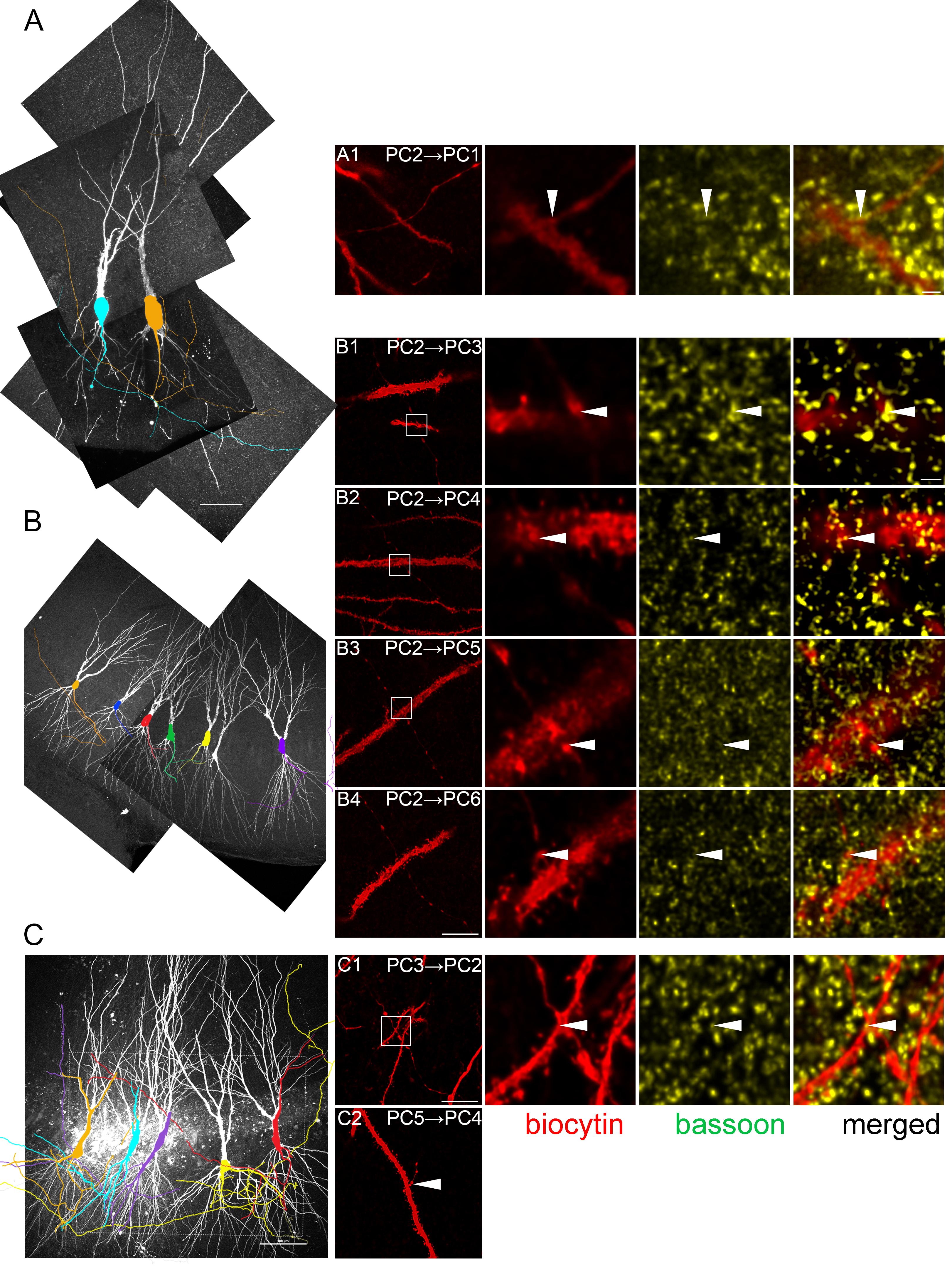

Supplement: Figure 1-2 — Identification of contact sites between axons and dendrites of biocytin-labeled CA3PCs in fluorescence-stained samples together with immunolabeling for synaptic protein, bassoon. Scale bars, for overview images: 50 µm, for maximal intensity projection images: 10 µm, for high magnification confocal images: 1 µm. Download Figure 1-2, TIF file. [file jneuro-45-e0102252025-s002.tif]

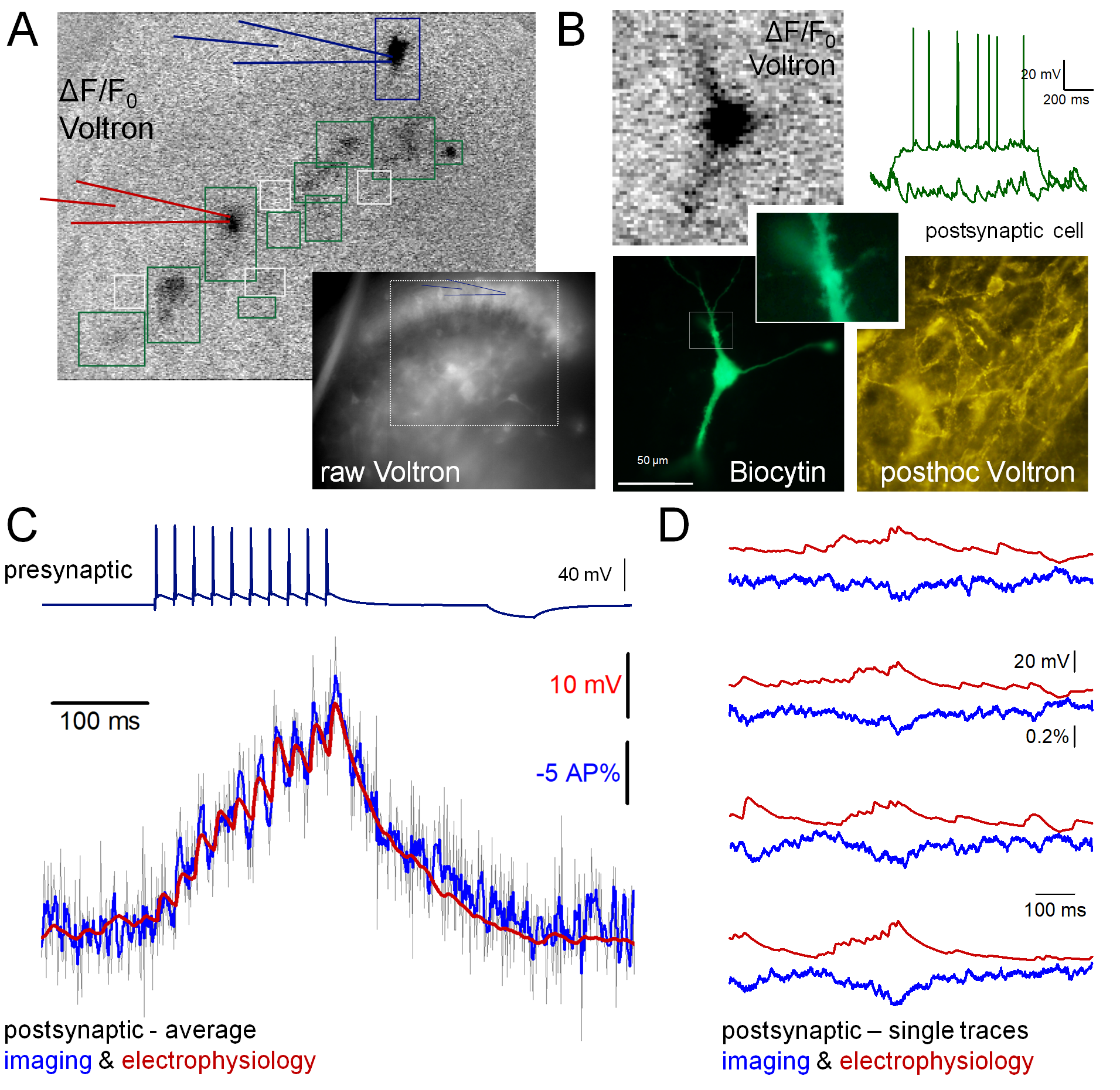

Supplement: Figure 2-1 — A. To test the sensitivity of Voltron imaging, we took advantage of the properties of excitatory synaptic connections between dentate gyrus granule cells and hilar mossy cells. Granule cells innervate mossy cells with a multi-release site giant synapse, which shows frequency dependent facilitation. Thus, it allows testing both smaller and larger responses in the same connection. Additional advantages of imaging in the hilus include the sparsity of neurons, the large size of mossy cells and large spontaneous synaptic potentials. B. The biocytin labelling by the recording pipettes allowed us to identify both neurons, the Voltron signal in their membranes and a putative synaptic contact in posthoc anatomical examinations. C. A single granule cell was stimulated with 10 APs at 50 Hz using patch clamp recording. The overall kinetics and amplitudes of the facilitating responses were faithfully followed by the Voltron imaging. Average of 19 traces. D. In addition to the evoked unitary responses, the largest individual spontaneous synaptic events could also be observed in individual traces with the help of the simultaneous patch clamp recordings. From these observations we concluded that Voltron imaging is sufficient for detecting unitary synaptic responses between identified neurons when multiple trials are averaged. Download Figure 2-1, TIF file. [file jneuro-45-e0102252025-s003.tif]

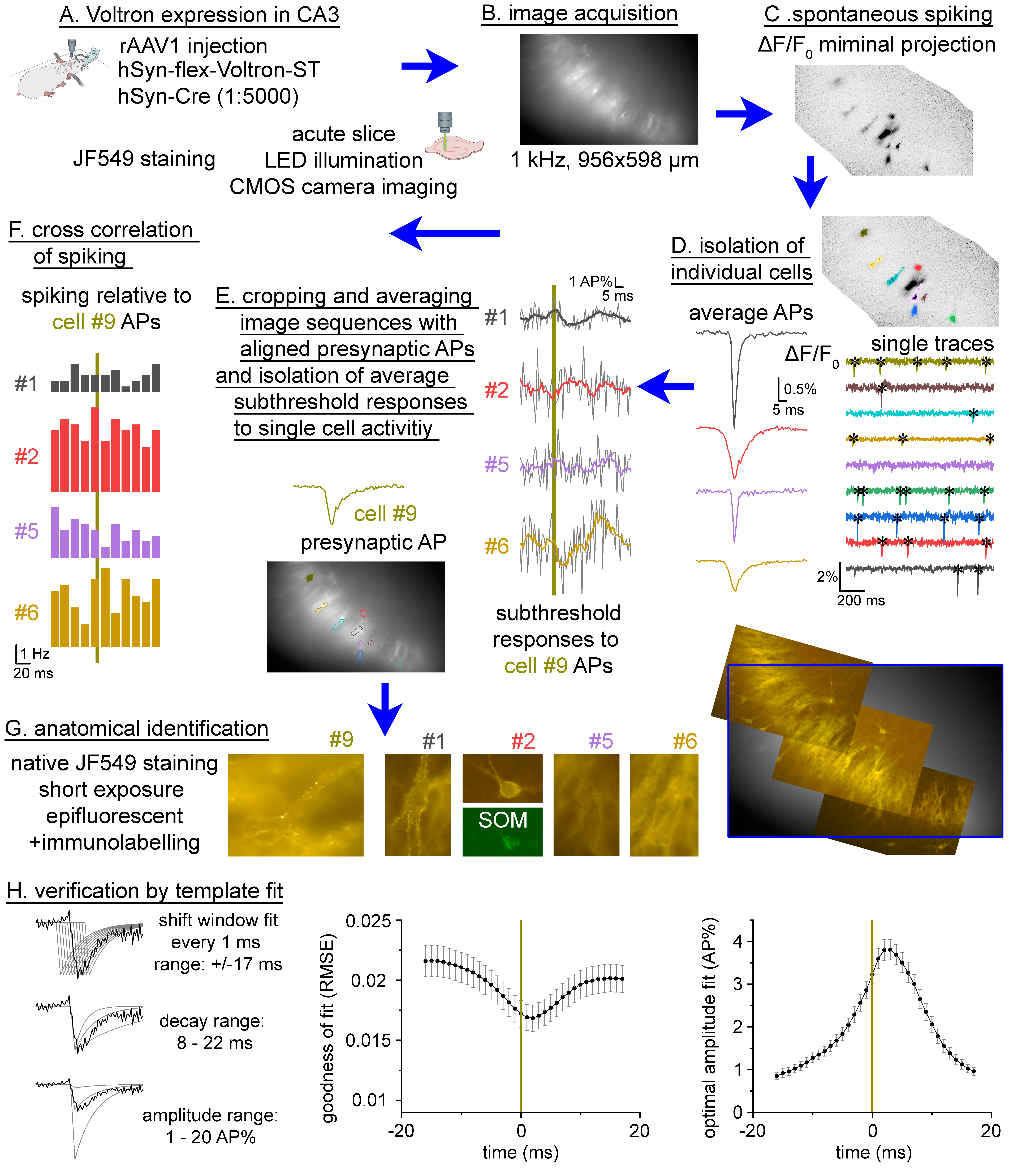

Supplement: Figure 2-2 — Workflow of detecting monosynaptic connections using spontaneous spiking and temporally aligned Voltron imaging. A. Sparse Voltron was expressed by a mixture of two AAVs. Acute slices were prepared 5-9 weeks after virus injection and incubated with Janelia Fluor 549 HaloTag (JF549). B. Imaging was performed at 1 kHz rate using a CMOS camera and LED light source. C. Identification of spiking neurons. D. The timing of each individual APs was determined (asterisk) and short videos were calculated in which spiking of a neuron is temporally aligned. E. Subthreshold signals from other cells were analyzed. F. Spike timings and their cross-correlations were also used to identify excitatory connections. G. After the imaging, posthoc anatomical examinations were performed to identify the type of spiking neurons. H. Shift window fitting. Left traces show parameter ranges that were allowed to change during fitting. Each average trace was repeatedly fitted with these variable EPSP waveforms in every frame. Graphs in the middle and right show the average parameters obtained using the variable fits for previously identified monosynaptic connections between CA3PCs. Notice that the EPSP-waveform fit was the most optimal 2-3 frames after the presynaptic AP, as shown by the lowest RMSE and highest amplitude. Download Figure 2-2, TIF file. [file jneuro-45-e0102252025-s004.tif]

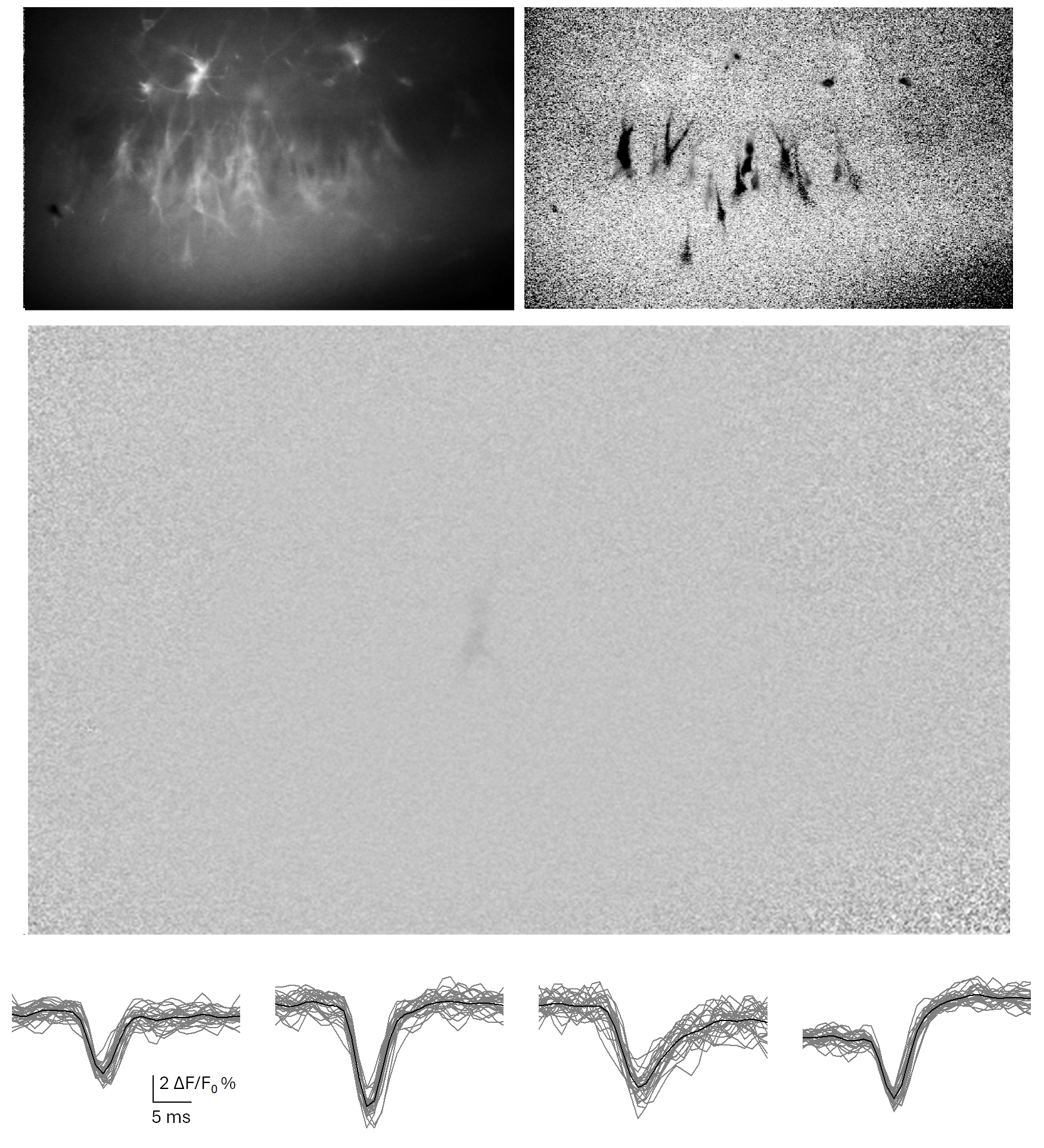

Supplement: Figure 2-3 — Top left: Raw fluorescence image of Voltron-labeled CA3 neurons. Middle: ΔF/F0 images of average spontaneous APs from 20 cells shown as a video. Top right: Minimal intensity projection of ΔF/F0 highlights all active neurons in this field view during the experiment. Bottom: 25 representative individual APs from four different neurons and their averages. Notice that due to the jitter following the peak detection of the 1 kHz signals, the average APs are slower and smaller than most individual APs. Download Figure 2-3, TIF file. [file jneuro-45-e0102252025-s005.tif]

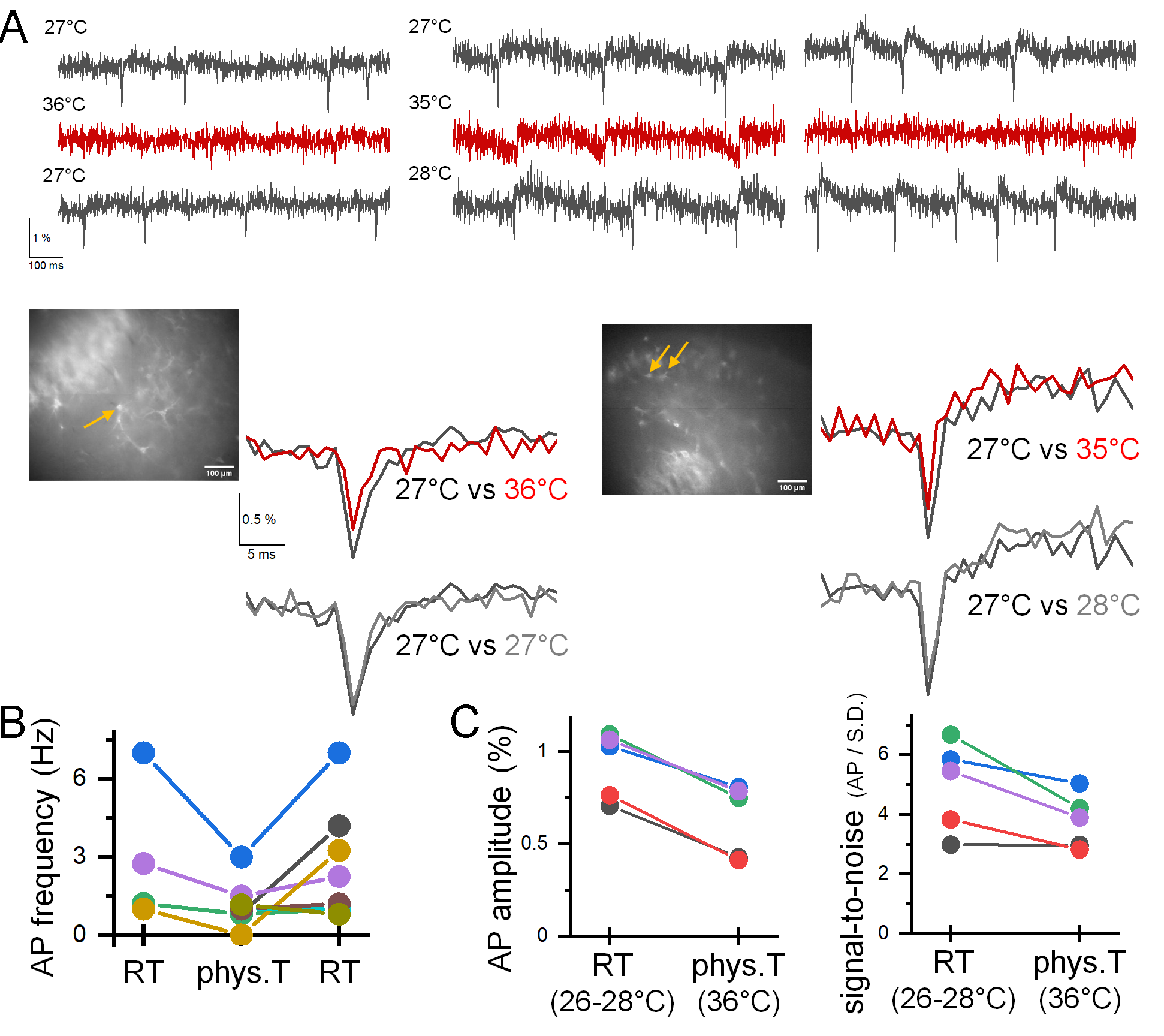

Supplement: Figure 2-4 — A. Temperature dependence of spontaneous AP detection by Voltron imaging in individual neurons. Two example experiments in which temperature in the chamber was raised to 35-36°C and subsequently returned to room temperature, while the same neurons were imaged. Example neurons are indicated by arrows. B. Summary data showing the frequency of detected APs in single neurons in different temperatures. Note that the lower number of APs may reflect either decreased firing or decreased detectability. Each color represents a single neuron. C. Properties of Voltron signals of spontaneous APs at different experimental temperatures. Connected symbols indicate individual neurons (n = 3 slice experiments). Download Figure 2-4, TIF file. [file jneuro-45-e0102252025-s006.tif]

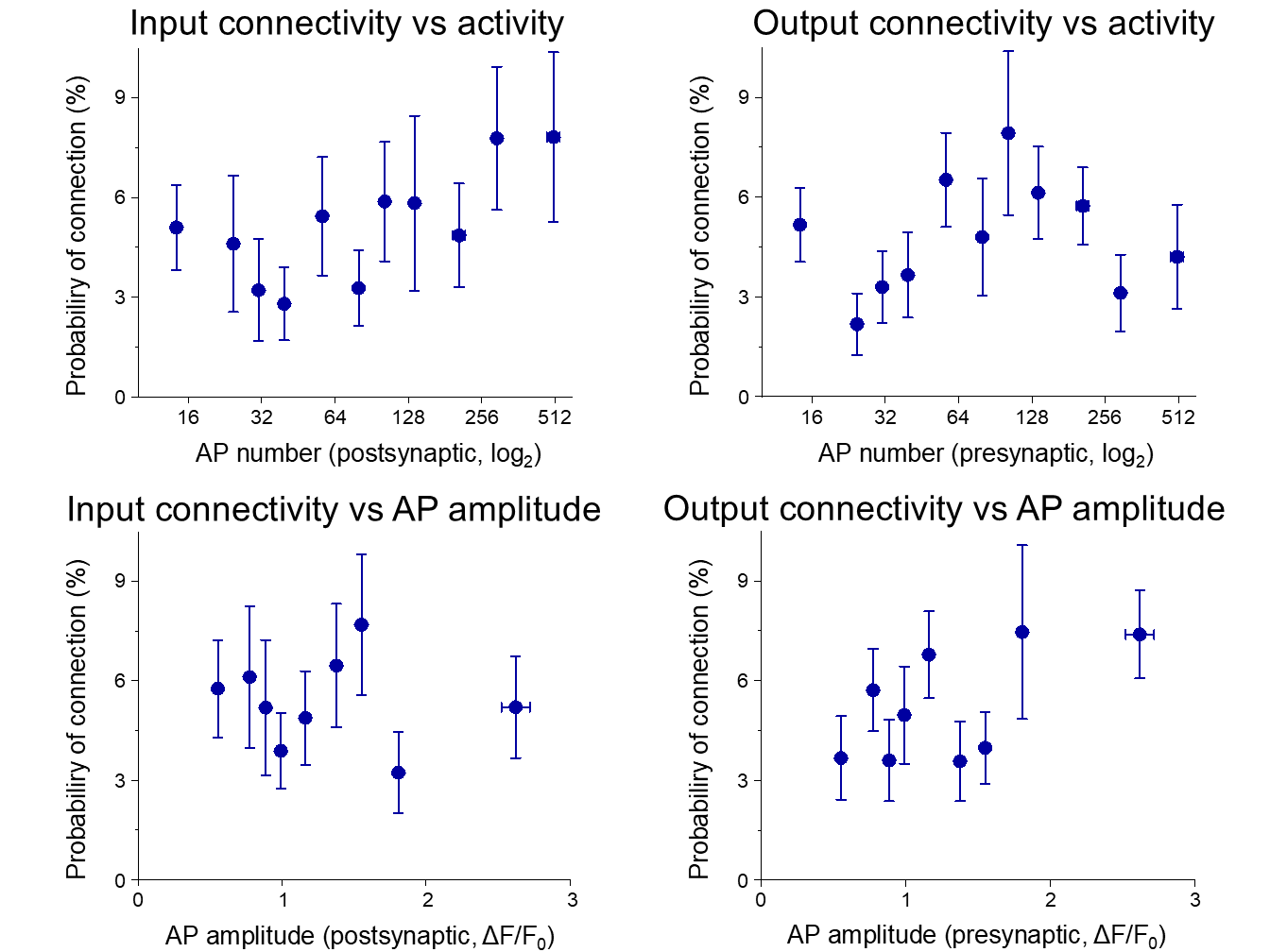

Supplement: Figure 3-1 — The number and amplitude of APs in CA3PCs did not show correlations with their input and output connectivity, suggesting that the detected connectivity rates did not depend on neuronal activity in our experimental conditions. Download Figure 3-1, TIF file. [file jneuro-45-e0102252025-s007.tif]

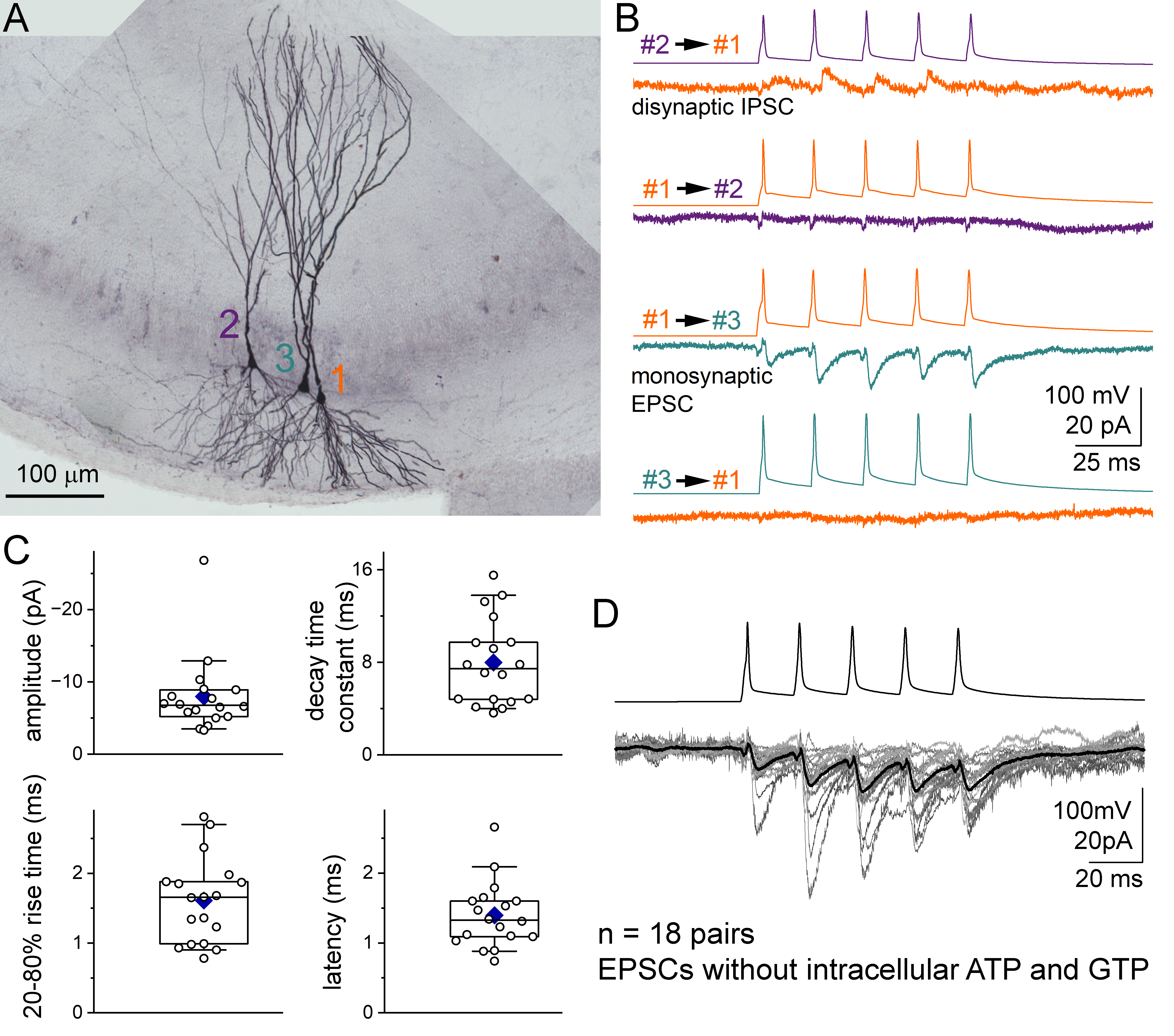

Supplement: Figure 4-1 — Electrophysiological characterization of monosynaptic connections between CA3PCs recorded without ATP and GTP. A. An example experiment demonstrating sequential paired recordings from three pyramidal cells. Collated light micrographs of biocytin-filled CA3PCs processed using DAB staining. B. The bottom traces show responses evoked by a short 50 Hz train. Postsynaptic cells were voltage-clamped at -50 mV. In this experiment, cell #2 elicited outward disynaptic IPSC responses in cell #1 (first trace pair), whereas cell #1 evoked a monosynaptic EPSC in cell #3 (third trace pair). C. Summary graphs of EPSC parameters. Open circles represent data from individual connections (n = 18 pairs), blue diamonds indicate mean values, and horizontal lines denote the median value. Boxes span the interquartile range (25%–75%), while whiskers indicate the 10%–90% range. D. Average monosynaptic EPSCs in 18 CA3PC-CA3PC pairs recorded without ATP and GTP (gray traces) and their average (black trace). The top trace displays the average of all presynaptic AP traces. Download Figure 4-1, TIF file. [file jneuro-45-e0102252025-s008.tif]
